# Polybetaines in Biomedical Applications

**DOI:** 10.3390/ijms22179321

**Published:** 2021-08-28

**Authors:** Stefania Racovita, Marin-Aurel Trofin, Diana Felicia Loghin, Marius-Mihai Zaharia, Florin Bucatariu, Marcela Mihai, Silvia Vasiliu

**Affiliations:** “Petru Poni” Institute of Macromolecular Chemistry, 41A Grigore Ghica Voda Alley, 700487 Iasi, Romania; stefania.racovita@icmpp.ro (S.R.); marin.trofin@icmpp.ro (M.-A.T.); diana.loghin@icmpp.ro (D.F.L.); zaharia.marius@icmpp.ro (M.-M.Z.); fbucatariu@icmpp.ro (F.B.); marcela.mihai@icmpp.ro (M.M.)

**Keywords:** polycarboxybetaine, polysulfobetaine, polyphosphobetaine, antifouling, antimicrobial, drug delivery systems

## Abstract

Polybetaines, that have moieties bearing both cationic (quaternary ammonium group) and anionic groups (carboxylate, sulfonate, phosphate/phosphinate/phosphonate groups) situated in the same structural unit represent an important class of smart polymers with unique and specific properties, belonging to the family of zwitterionic materials. According to the anionic groups, polybetaines can be divided into three major classes: poly(carboxybetaines), poly(sulfobetaines) and poly(phosphobetaines). The structural diversity of polybetaines and their special properties such as, antifouling, antimicrobial, strong hydration properties and good biocompatibility lead to their use in nanotechnology, biological and medical fields, water remediation, hydrometallurgy and the oil industry. In this review we aimed to highlight the recent developments achieved in the field of biomedical applications of polybetaines such as: antifouling, antimicrobial and implant coatings, wound healing and drug delivery systems.

## 1. Introduction

The challenges that always appear in the medical and pharmaceutical fields have determined that various research groups turn their attention to the development of new materials that, through design and properties, lead to finding solutions regarding the improvement of human health and compliance of the patients.

In recent years, researchers in the medical and pharmaceutical fields have generally focused on the following findings:

- The discovery of new drug delivery based on micro and nano-sized particles that have the ability to respond to stimuli, to carry biologically active targeting principles, to treat cancer or have a multifunctional role in the delivery of therapeutic genes.
- The use of polymeric materials for diagnosis, therapeutic and biomedical applications, particularly in tissue engineering.

In the last period, zwitterionic polymers have gained increased interest as biomaterials due to their antifouling and antimicrobial properties, easy functionalization and flexibility of structural design [1]. The family of zwitterionic polymers includes a wide range of macromolecules, from natural ones such as proteins and nucleotides to those of synthesis and semisynthesis compounds [2]. Depending on the charge distribution mode, the polyzwitterions can be polyampholytes and polybetaines [3,4]. 

**Figure 1:**
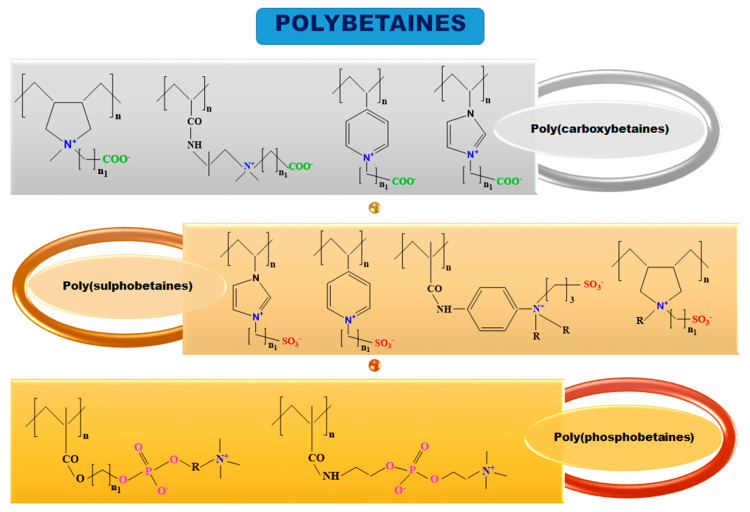
Types of polybetaines and some of their chemical structures.

The difference between polyampholytes and polybetaines consists in the charge position, as follows: polyampholytes are polymers that have positively:negatively charged groups (1:1) situated on different structural units [5] while the polybetaines refer to the polymers that have both anionic and cationic groups on the same monomer unit, separated by an alkyl chain with variable length [6]. In polybetaine structure the cationic group is represented by quaternary ammonium groups, while the anionic groups can be carboxylate groups [poly(carboxybetaines)] (PCB), sulfonate groups [poly(sulfobetaines)] (PSB) or phosphate/phosphinate/phosphonate groups [poly(phosphobetaines)] (PPB) (Figure 1).

In literature, we can find two main methods to obtain polybetaines:

- Polymerization of zwitterionic monomer using several polymerization techniques, such as: free radical polymerization (FRP), controlled radical polymerization (CRP), atom transfer radical polymerization (ATRP), group chain-transfer polymerization (GTP), reversible addition fragmentation transfer method (RAFT), distillation-precipitation polymerization (DPP) and reversible-deactivation radical polymerization (RDPR) [7,8,9,10]. By this method polymers with 100% betaine groups are obtained but the polymerization of zwitterionic monomer method has the disadvantage that the molecular mass of synthesized polybetaines cannot be determined accurately by gel permeation chromatography (GPC) and high performance liquid chromatography (HPLC) measurements because polybetaines show strong interactions with the materials found in the chromatographic columns [8].
- Betainization of polymer precursor containing tertiary ammonium groups using polymer-analogous reactions [11]. In this case, the polybetaines are easy to be characterized, being able to obtain the polymers with varied and well-defined chemical structures, but due to the neighbouring groups effects and the complex reactions during the chemical functionalization, the polymer-analogous transformations cannot occur with a yield of 100%.

Additionally, the grafted polymerization strategy (grafted from, grafted to and grafted through) and click chemistry method are often used to obtain the polybetaines, especially for surface coatings [12,13,14]. 

The diversity of chemical structures of polybetaines are given by: the different nature of ionic groups (carboxylate, sulfonate or phosphate/phosphine/phosphonate groups); the length of the alkyl spacer separating the anionic group from the cationic one; the chemical structure of the starting monomer or polymer; the capacity of zwitterionic polymer to switch between zwitterionic and non-zwitterionic forms [15,16,17,18]. 

Since 1957–1958, when the first polybetaines such as poly(carboxybetaine) (1957) and poly(sulfobetaine) (1958) were discovered by Landenheim and Horawetz [19] and Hart and Timmerman [20], respectively, and until today, these polymeric materials have undergone continuous development, being studied in terms of structure and properties but mainly they have been developed to be used in various areas of great interest.

**Figure 2:**
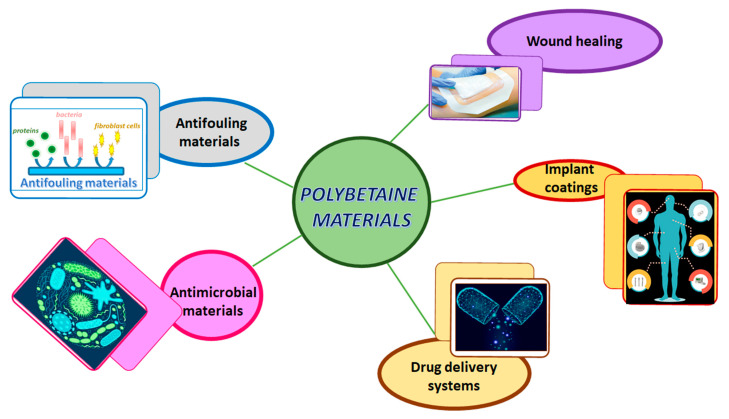
Biomedical applications of materials based on polybetaines.

Due to their unique properties such as antifouling and antimicrobial properties, low cytotoxicity, high biocompatibility, negligible immunogenicity, in vivo stability and long circulation time, the polybetaines can be used in some medical and pharmaceutical applications as shown in Figure 2.

The idea behind this review was to present the importance of polybetaines in the biomedical field, because there is just a small number of papers devoted to this purpose. In literature many researches have been directed towards the synthesis and properties of polybetaines. Zwitterionic materials, by default polybetaines, can improve biocompatibility, promote cellular uptake of traditional drugs, can be responsive to stimuli and can possess tumor targeting capacity. For this reason, in this review some of the directions of possible usage of polybetaines in the biomedical field are presented, namely: antifouling and antimicrobial coatings, drug delivery systems, wound healing and implant materials.

## 2. Biomedical Applications

### 2.1. Antifouling Materials

Biofouling represents the accumulation of microorganisms, plants, algae or small animals on various surfaces, while antifouling represents the repelling of protein adhesion through polymer hydration [17,21].

For this reason in biomedical applications (medical implants, drug delivery systems and biosensors) there is a considerable need for innovative antifouling coatings which can resist nonspecific protein adsorption and cell attachment [22]. The zwitterionic polymers keep electric neutrality with an equal number of positive and negative charged groups. This mix of contrary charged groups grants the high hydrophilicity of the polymer, preventing nonspecific protein adsorption and unwanted bacterial adhesion. Nowadays, there are not many materials that can meet these expectations, especially when challenged by real-world complex media, but there are many—ongoing research projects regarding zwitterionic polymers with—high wettability and antifouling properties [23].

Regarding the design of bioinert polymers, researchers have been inspired by—natural models such as the blood-contacting surface of the vascular endothelial cells. The key is to reproduce good surface hydration because it grants biocompatibility and protection against undesirable protein adsorption [24]. Most often, zwitterionic polymers are seen as a substitute for the well-known poly(ethylene glycol) (PEG) to block protein adsorption as well as to limit bacterial and mammalian cell adhesion [25]. However, the drawback of PEG is its tendency to auto-oxidize in the presence of oxygen and transition metal ions such as Cu(II) and Fe(III). Additionally, the PEGylation of proteins significantly reduces their bioactivity and triggers antibody production. Contrarily, polyzwitterions increase protein stability and maintain or even improve protein bioactivity [26]. Therefore, during computer simulations and laboratory experiments, zwitterionic polymers proved to be similar or even superior to PEG-based hydrophilic polymers regarding their resistance against protein adsorption, cell and bacterial attachment [27].

The connection between interfacial waters and antifouling properties has been studied by multiple research groups [28,29]. PEG uses a “steric repulsion” and a hydration layer generated by hydrogen bonding, which prevents protein adsorption [30]. Experimental evidence of the hydration layer was provided by Tanaka and Sackmann who furthermore classified PEG interfacial waters into three classes: nonfreezing water (which did not crystalize at −100 °C), freezing bound water (crystalizes below 0 °C) and free water (crystalizes at 0 °C) [31]. As demonstrated by vibrational sum-frequency generation, interfacial water molecules are present in polymeric self-assembled monolayers [32]. The highly hydrated polymer shows antifouling properties and as a result of this strong correlation, decreasing the surface hydration conducts to a lower protein repelling capacity. Therefore hydrophilic materials, namely mannitol and PEG, frequently switch from antifouling to fouling as a consequence of changes in surface hydration. 

In comparison, polybetaines can bind water stronger than the hydrophilic PEG materials, since electrostatically induced hydration is better than hydrogen bonding hydration. Due to the strong ionic solvation, zwitterionic polymers are excellent biocompatible antifouling materials. On the other hand, the antifouling capacity greatly relies on molecular weight, packing density and temperature [23].

Some structures of betainic monomers and polybetaines with antifouling properties are presented in Table 1.

Wang and colab [37] developed a cross-linked sulfobetaine polymer network (ZPN) coatings based on 2-[(methacryloyloxy)ethyl]dimethyl-(3-sulfopropyl)ammonium hydroxyde (MEDSAH) and poly(ethylene glycol) dimethacrylate (PEGDMA).

The ZPN coating was obtained by spin-coating/drop-casting the polymer solution onto 3-aminopropyl triehoxysilane modified silicon or silicon nitride surface and displayed excellent protein repellence and self-healing properties. An important property of this coating is that when it is mechanically damaged it can be repaired by contact with water within 1 min. In addition, both the mechanical properties and the antifouling properties are repaired after healing. 

A number of studies have shown the advantages of polybetaines when they either fully coat membranes or when membranes are produced from mixtures of polybetaines [38,39,40]. Membranes are known to be important for certain medical applications such as dialysis. The ideal surface of an artificial blood purification membrane should be hemocompatible and have high performance durability; it should not adsorb proteins or cells, but should still have high permeability in the desired solute size range [41]. 

A random copolymer was synthesized by reacting hydrophilic N,N-dimethyl-N-methacryloxyethyl-N-(3 sulfopropyl (DMMSA) with hydrophobic butyl methacrylate (BMA) through a radical polymerization [38]. The obtained sulfobetaine copolymer was mixed with polyethersulfone (PES) to prepare an ultrafiltration membrane for bovine serum albumin (BSA) separation. The antifouling property of the membrane was improved with increasing concentration of sulfobetaine copolymer in the membrane casting solution [38].

Lee and colab. [39] synthesized via plasma-induced surface polymerization the polypropylene (PP) fibrous membranes grafted with poly(sulfobetaine methacrylate). This study demonstrated that the betaine membrane exhibits antifouling character.

Ye and colab. [40] mixed cellulose acetate membrane with poly(2-methacryloyloxyethyl phosphorylcholine (MPC)–co-butyl methacrylate (PMB30) copolymer and obtained membranes that were mechanically resistant and adsorbed less protein and platelets than membranes made of pure cellulose acetate or polysulfone.

### 2.2. Antimicrobial Materials

The use of the polymeric materials with antimicrobial properties is an extremely necessary solution if we consider the fact that the microorganisms undergo mutations, making them resistant to the action of the existing pharmaceutical products on the market. Today, there are about 2500 microorganisms that cause infectious diseases, which are responsible for 60–70% of total mortality.

The nosocomial infections or healthcare-associated infections are a major problem faced by healthcare systems in both developed and low-income countries.

The term nosocomial infection refers to any systemic or localized condition that is the result of an interaction with an infectious agent or toxin [42,43]. 

According to the Centers for Disease Control and Prevention (CDC), nosocomial infections are caused by different types of pathogens: bacteria (*Staphylococcus aureus, Pseudomonas aeruginosa, Escherichia coli, Klebsiella, Proteus, Enterobacter, Clostridium difficile, Salmonela, Streptoccoci*), some fungi (*Candida albicans, Aspergillus spp., Nocardia, Pneumocystis carinii, Cryptococcus neoformans, Cryptosporidium*) and viruses [Respiratory syncytial virus, Cytomegalovirus, Human immunodeficiency virus (HIV), Ebola, rotavirus and enteroviruses, Influenza A (subtypes H2N2 and H3N3), hepatitis and herpes viruses].

To create a new material with antimicrobial properties, it is necessary to know the structure of the microorganisms and how they interact with the given materials or with the low molecular weight compounds (antibiotics) developed until now.

From structural point of view, the component of the bacterial cell can be divided into two categories:

- Intraparietal: the cytoplasmic membrane, mesosomes, cytoplasm, nucleotide, ribosomes, magnetosomes [44,45,46,47].
- Extraparietal: capsule, mucus layer, glycocalyx, flagella, fimbriae and pili.

The bacterial cell is delimited on the outside by the cellular wall which is a complex structure of variable thickness between 15–35 nm consisting of peptidoglycans. Peptidoglycan is a heteropolymer containing a glycan portion (linear chains of alternating N-acetyl-glucosamine and N-acetyl muramic acid, linked together by β (1→4) bonds) and a peptide component, which has in its structure the D-aminoacids (L-alanine, D-isoglutamic acid, L-lysine or diaminopimelic acid and D-alanine) which have the role to protect the cell wall from the destructive action of the peptidases. The D-amino acids from the peptide component bind to the muramic acid residues on the glycan chains via amide links creating a porous three-dimensional network resembling to a small mesh [48,49]. 

Depending on the structure of the cell wall, bacteria are classified into Gram-positive and Gram-negative bacteria. The cell walls of these types of bacteria differ by the ratio, quantity, nature and structural arrangement of the components [50,51,52,53].

Cytoplasmic membrane (7.5–10 nm) represents the structural formation found between the inner surface of the cell wall and the cytoplasm and consists of proteins (integrated and surface), phospholipids and carbohydrates (glycoproteins and glycolipids). 

In the synthesis of substances with antimicrobial properties (antibiotics and macromolecular compounds) the starting point was the cell wall structure of microorganisms and methods were sought to obtain those structures that lead to the weakening of the cell wall and finally to the destruction of the pathogen agent. Thus, the following targets were pursued:

- Biosynthesis of the cell wall. Considering that the cell wall is the one providing the stability and mechanical resistance, attempts have been made to develop antimicrobial materials that would prevent the main component (peptidoglycan) to form the crosslinks in order to weaken the cell wall resistance and ultimately to lead to the destruction of the bacteria.
- Protein synthesis. In this case, the synthesized molecules turned out to be inhibitors that blocked protein biosynthesis especially at the ribosome level.
- DNA replication and repair [54].

The development of new antimicrobial materials that are capable to prevent bacterial adhesion and biofilm formation by killing or inhibiting the growth of microorganisms located on different surfaces and in the environment represents an important task for the scientific world [55]. 

**Figure 3:**
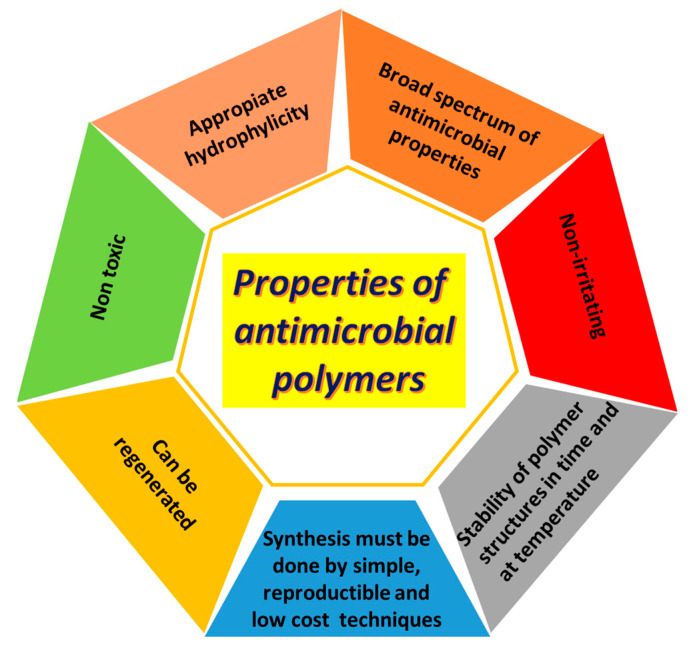
Requirements for an effective antimicrobial polymers.

In order to efficiently prevent the attachment and proliferation of bacteria, the antimicrobial materials should meet certain requirements that are shown in Figure 3 [56,57].

Until now, five types of antimicrobial materials are specified in literature:

- Polymeric biocides are the polymers that have bioactive repeating units with antimicrobial activity (amino, carboxyl and hydroxyl groups) [58,59].
- Biocidal polymers are the polymers that contain quaternary ammonium, phosphonium, tertiary sulfonium and guanidinium groups as cationic biocides. The mechanism of antibacterial action can be described as follows: adsorption onto the bacteria cell surface that is usually negatively charged; diffusion through the cell wall; binding and disruption of cytoplasmic membrane; release the component (electrolyte and nucleic acids) of cytoplasmic membrane and finally the death of the bacteria cell [60,61].
- Biocide releasing polymers represent a polymeric carrier for biocide molecules that can be covalently linked or physically entrapped [62].
- Bioactive peptide [63,64,65].
- Antimicrobial surfaces can be achieved by using two methods: (a) chemical methods that include surface coatings and modification of the surface chemistry through functionalization, derivatization or polymerization; (b) physical methods which are responsible for the modification of the structure architecture [66,67].

In the case of polybetaines the chemical method is often used to obtain the polybetaine polymer-based surface coatings (surface brushes and adhering membrane) [55,68,69]. There are a variety of polymer coating methods as follows: drop-casting coating method [70], cast-coating [71], tape casting [72], spraying method [73] and spin coating technologies [74].

The surface coatings can be categorized into three types:

- Contact killing surfaces that are functionalized with bactericide (disinfectants, antiseptics or antibiotics) through covalent linkages, physical absorption or coordination bonds [75].
- Anti-adhesion/bacteria repellent surfaces. In this case the surface is coated with hydrophilic polymers that can prevent bacteria accumulation and proliferation by establishing steric repulsion through surface hydration or charge repelling [76].
- Killing-release surfaces [77,78]. This is a relatively new approach designed to solve the problems of infections that appear in clinical surgery and implantation operations, consisting of a combination between antifouling and surface bactericidal properties.

Some of these antibacterial surfaces (passive-defense antibacterial surfaces) are ineffective against bacteria once they adhere onto the surface, while another bacterial surfaces (active-attack antibacterial surfaces) can lose their antimicrobial activity due to the accumulation on the surface of dead bacteria and debris [61]. 

In this context, the smart material which possess both antifouling and bactericidal properties at the same time as well as the switching capacity upon external stimulus such as, pH, light, ions, electric field and CO2 plays an important role in biological systems [17,79,80,81,82].

Regarding the switching behaviour, a polybetaine is susceptible to protonation in acidic conditions. Since bacterial cells are usually negatively charged, they can be electrostatically attracted to the protonated polyzwitterion, for further release in high pH conditions [25]. Similar shifting behaviour is specific for zwitterionic precursor surfaces such as poly(carboxybetaines)-esters (PCB-esters). In the ester form, the polymer is basically a quaternary ammonium compound with wide antibacterial activity. Bacteria contacting this surface are killed and build upon the polymeric coating, significantly reducing the antimicrobial activity and triggering the immune response. This build-up of dead microorganisms is released upon hydrolysis. The surface switches to a nontoxic, anti-fouling state, preventing further bacterial attachment.

The hydrolyzable PCB synthesized (101 dins ultralow) in the ester form killed more than 99.9% of the *Escherichia coli* strain K12 in one hour and upon hydrolysis, 98% of the attached dead bacteria was released revealing an ultralow fouling polybetaine surface [83]. These switchable polybetaine ester-based coatings are the answer to an old biomedical issue because, during surgery, it inactivates bacterial cells which establish contact with it, preventing wound infection. After hydrolysis, it turns biocompatible and low fouling preventing protein and bacterial adhesion, prolonging lasting time of the implant [23].

The only downside of this coating is the fact that it can only switch once, which is not enough for certain requests such as coating the surgical equipment or the surfaces in the operating room. For such applications, a reversible lactonization reaction was used, ensuring the renewal of the coating. In the cyclic cationic form, the polymer has high antimicrobial activity. In the presence of water, it occurs the ring-opening reaction revealing the polybetaine, low fouling state. To renew the coating it is necessary to dehydrate it, at low pH conditions, back to the lactone state. Such switchable materials prove chemical stability over multiple cycles [25].

Ion-responsive smart materials based on poly(3-(1-(4-vinylbenzyl)-1-H-imidazol-3-ium-3yl)propane-1 sulfonate) brushes obtained via the surface-initiated ATRP exhibited reversible surface wettability switching between water and salt solutions. In water, betaine brushes induced higher protein absorption, higher surface friction and lower surface hydration compared to those in salt solutions, being able to switch reversibly and repeatedly between protein capture/release and surface wettability in a controllable manner [18,84].

Smart antibacterial surfaces consisting of two polymer brushes with two layer architecture were obtained by Yang and colab. [78] as follows:

- Upper layer bactericidal brushes are based on poly[trimethyl amino)ethyl methacrylate chloride] (polyMETAC) or poly[2-(tert-butylamino)ethyl methacrylate] (polyTA).
- Background layer consisting of salt-responsive polybetaine brush based on poly(3-(dimethyl(4-vinylbenzyl)ammonio)propyl sulfonate (polyDVBAPS).

By combining these two layers, salt-responsive surfaces which can reversibly kill and release bacteria (*Escherichia coli* and *Staphylococcus aureus*) in response to the switch between water and salt solution were developed. Such systems displayed high killing efficiency (93%) and highly efficient regeneration capability by releasing in NaCl solution of ~ 90% of attached bacteria having great potential to be used in biomedical and industrial fields [78]. 

**Figure 4:**
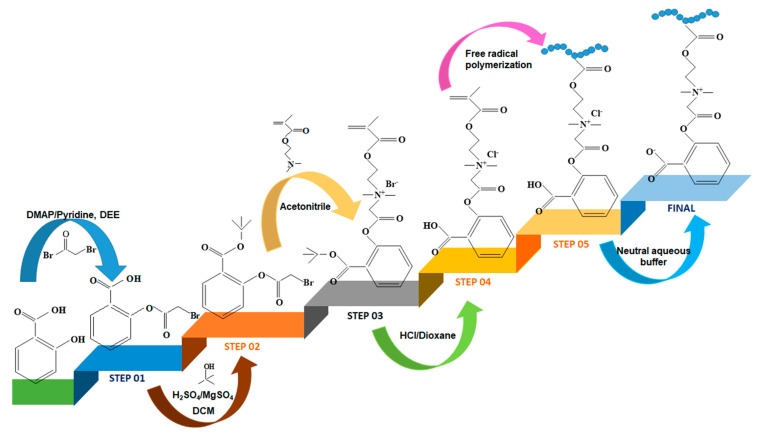
Schematic representation for the synthesis of PCBSA polymer (adapted from Ref. [85]) DMAP—4-(dimethylamino) pyridine; DEE—anhydrous diethyl ether; DCM—dichloromethane).

The combination between antifouling and antimicrobial properties was used by Jiang and Mi [85] to prepare a betaine hydrogel based on polymer drug-conjugate namely poly(2-(2-(92-(methacryloyloxy)ethyl)dimethyl ammonia)acetoxy) benzoate (PCBSA) (Figure 4).

This betaine hydrogel is able to keep the surface free from bacteria and simultaneously inhibit bulk bacteria growth in a controllable manner as well as can either used as a stand-alone drug delivery systems or as a coating for other biomedical devices (catheters, implants and artificial bones).

In addition, a series of polymethacrylic and polyimine sulfobetaines and carboxybetaines have been studied for their antibacterial activity against Gram-positive (*Staphylococcus aureus*) and Gram-negative (*Pseudomonas aeruginosa* and *Escherichia coli*) bacterial strains [16,86].

Horiguchi and colab. [87] developed a highly selective diagnostic system based on gold nanoparticles covered with sialic acid/sulfobetaine hybrid surface for individual counting of Influenza A H1N1 subtype using sensitive pulse sensing.

### 2.3. Drug Delivery Systems

Drug delivery is a way of administering a biologically active principle through different routes (oral, parenteral, sublingual and buccal, rectal, vaginal, ocular, otic, nasal, cutaneous and transdermal) in order to obtain an adequate therapeutic effect in humans and animals [88]. The increasing research in the medical and pharmaceutical fields is directed towards finding new materials that can be used in the design of new drug delivery systems. These materials must be stable in different pH media, biodegradable and biocompatible, have negligible immunogenicity, present high encapsulation and release efficiency and show no toxic effects. Table 2 shows the structures of the most representative polybetaines used in preparation of drug delivery systems.

#### 2.3.1. Drug Delivery Systems in Cancer Therapy

Cancer is a term used to define malignant conditions in which abnormal cells multiply in an uncontrolled and continuous way, and can invade the surrounding healthy tissue. Abnormal cells come from any tissue in the human body and can occur anywhere in the body.

Clinically, cancer is a large group of diseases that vary in their mode of onset, growth rate, diagnosis, detectability, potential for invasion, metastasis, response to treatment and prognosis. Unfortunately, cancer remains one of the biggest health problems facing humanity. For this reason, a lot of research is being directed towards the development of anti-tumor drug delivery systems that improve therapeutic efficacy and limit side effects as much as possible. Below, some studies that can be used in cancer therapy based on betaine materials will be presented.

Among the phosphobetaine polymers, phosphorylcholine (PC) is used to obtain biomimetic materials because it is a component (the hydrophilic polar head group) of some phospholipids that plays an important role in cell membranes. Thus, studies have shown that nanostructures containing phosphorylcholine penetrate living cells through fusogenic interaction with plasma membranes [89].

In 1990, Ishihara and colab. [90] synthesized a phosphorylcholine monomer, namely 2-methacryloyloxyethyl phosphorylcholine (MPC), which was subsequently used to obtain a variety of polymers with phosphobetaine structure. This research group continued their studies, and in 2003 they developed the first phosphorylcholine-based drug delivery system, which was designed to carry paclitaxel (PTX) [91]. This drug delivery system improved both the water solubility of PTX and its stability in the bloodstream due to the antifouling property of the phosphorylcholine monomer.

In 2007, Hsiue and colab. [92] reported the use of the MPC monomer and the poly (D,L-lactide)-bromine initiator to synthesize the diblock copolymer poly(2-methacryloyloxyethyl phosphorylcholine)-block-poly(D,L-lactide) (PMPC-b-PLA) (Table 3) by atom transfer radical polymerization method.

A few years later, Liu and colab. [93] synthesized PMPC-PLA-based vesicles to encapsulate two anticancer drugs (hydrophobic doxorubicin (DOX) and hydrophilic doxorubicin hydrochloride (DOXHCl)) both inside the vesicles and in their membranes. In vitro release studies showed that the release of the two drugs was pH-dependent, i.e., faster drug release occurred at pH 5 compared to pH 7.4. This behaviour could be attributed to PMPC-PLA hydrolysis which results in morphological transformation from vesicles to micelles, leading to the release of the encapsulated drugs.

Further research by Wang and colab. [94] led to a biocompatible and pH-sensitive copolymer that was synthesized by bridging poly(2-methacryloyloxyethyl phosphorylcholine) block and poly(D,L-lactide) (PLA) block by a benzoyl imine linkage (Blink). Thus, biomimetic micelles based on PLA-Blink-PMPC copolymer (Table 3) were obtained and were then used as carriers for PTX delivery. The PTX-loaded micelles are quite stable at normal physiological pH, but decomposed over 6 h in the acidic tumor environment, leading to rapid drug release, thus having improved antitumor efficacy. It can therefore be concluded that the micelles can be used as biocompatible and intelligent drug delivery systems.

Various polymer-drug conjugates (also called prodrugs) have been developed for malignant tumor therapy. These have some advantages such as: maintaining the structure of the micelles, reducing the drug leakage during blood circulation and drug release directly into the tumor [95]. Ji and colab. [96] synthesized a prodrug based on phosphorylcholine (Table 3) by conjugating of DOX to 11-mercaptoundecyl phosphorylcholine via an acid-labile hydrazine linker (UnPC-*hyd*-DOX). This prodrug can self-assemble in core-shell micelles with high drug content (~57%) and can minimize nonspecific phagocytosis by macrophages. Compared to the prodrugs based on polyethylene glycol, the PC conjugated has some advantages such as: it facilitates penetration into cancer cells due to the PC coating; it shows much lower cardiotoxicity than free use of DOX and shows much slower blood clearance.

Another research of Wang and colab. [97] was also directed towards the preparation of a polybetaine PMPC-PLL (DOX) prodrug (Table 3) using poly(L-lysine) (PLL) as the functional segment for binding DOX via imine linkage. The PMPC-PLL (DOX) prodrug can self-assemble in micelles with core-shell structure in physiological medium, and when transferred to acidic medium (pH 5.5) the imine bonds are disrupted to reverse the surface charge, so as to contribute to endosomal escape and accelerated drug release.

Jin and colab. [98] developed DOX encapsulated pH-responsive polymeric micelles starting from a novel phosphobetaine copolymer poly(2-methacryloyloxyethyl phosphorylcholine-co-2-(4-formylphenoxy) ethyl methacrylate (poly(MPC-co-FPEHA) synthesized via RAFT polymerization. Then the phosphobetaine copolymer was converted into PMPC-*hyd*-TPE (Table 3) after conjugation with tetraphenylethene (TPE) via acid-cleavable hydrazine bond having the ability to self-assemble into spherical micelles that could load the anticancer drug (DOX) through hydrophobic interactions. Betaine micelles showed excellent physiological stability and high protein resistance due to the antifouling properties of PC having a huge potential as nano-platform for cancer theranostics.

Recent studies have also highlighted the use of sulfobetaines in drug delivery systems synthesis because these systems exhibit efficient loading of biologically active principles, high cellular uptake rate, low cytotoxicity and weak immune response. Wang and colab. [99] considering both the acid-labile characteristics of acetal segments and the charge-reversing effects of zwitterionic fragments developed a sulfobetaine-functionalized polyacetal dendrimer for antitumor drug delivery. These biocompatible DOX-loaded sulfobetaine dendrimers exhibited good stability in physiological medium, sustained drug release over a long period of time even under acidic conditions and remarkable cytotoxicity against cancer cells.

A biodegradable nanogel based on poly(sulfobetaine methacrylate) was made by one-step reflux precipitation copolymerization of sulfobetaine methacrylate (SBMA) and N,N′-bis-(acryloyl) cystamine (BAC) [100]. It was observed that DOX-loaded PSBMA nanogels exhibited the strongest growth inhibition effect on hypopharyngeal carcinoma.

Because poly(carboxybetaines) have been shown to have remarkable hydrating properties in different media and carboxylate groups of PCB can interact ionically with biomolecules containing amino groups (antibodies, ligands, fluorescent dyes), these polymers may be ideal candidates for the preparation of betaine materials with applications in biomedical field [101,102].

Gu and colab. [103] obtained ternary nanoparticles poly(carboxybetaine methacrylate) (PCBMA)(peptide dendrimer-modified carbon dots (CD-D)/doxorubicin (DOX))] based on peptide dendritic carbon dots (CDs) that have been used in cancer therapy. The polycarboxybetaine coating ensures the safe transport of nanoparticles PCBMA(CD-D/DOX) through the circulatory system due to their antifouling properties that give them the ability to resist nonspecific protein adsorption. The existence of acidic compartments and a high redox level inside tumor cells led to the destruction of PCBMA(CD-D/DOX) with the possibility to achieve both a rapid and efficient tumor-targeted release of the drug and its highly efficient intracellular trafficking toward nuclei. In addition, the excellent fluorescence properties of the CDs conferred PCBMA(CD-D/DOX) with fluorescence bioimaging function. This study showed that PCBMA(CD-D/DOX) exhibited remarkable antitumor activities both in vitro and in vivo, demonstrating much higher antitumor efficacy and fewer side effects than using free DOX.

A number of studies have shown that in order to increase the specific accumulation of drugs at tumor sites, different tumor components such as folic acids [104], mucopolysaccharides [105], peptides [106] or antibodies [107] have been introduced onto the surface of the carriers. Therefore, these specific target ligands were introduced into different betaine materials so that they could be recognized by the corresponding receptors on the surface of the tumor cells.

Armes and colab. [108,109] directed their research towards the synthesis using the ATRP method of a series ofpolybetaine block copolymers based on PMPC and poly[2-(diisopropylamino)ethyl methacrylate] (PDPA) (PMPC-b-PDPA) (Table 3). The pH-sensitive DPA blocks are protonated and therefore hydrophilic in acidic media, but when the solution is adjusted to physiological pH they become deprotonated. Therefore, this could lead to self-assembly of PMPC-b-PDPA micelles in the bloodstream, leading to loading of water-insoluble drugs by the hydrophobic PDPA core [109]. When the micelles are endocytosed in the cells, the PDPA blocks are protonated and therefore can no longer maintain the micelles structure resulting in rapid drug release into the cytoplasm. Adjusting the degree of polymerization of PMPC and PDPA leads to both regulation of drug release kinetics and their working morphology (micelles, vesicles, etc.) [110].

**Table 3:** Chemical structures of polybetaine materials used in cancer therapy.

	Chemical Structures of Polybetaine Materials	Ref.
1	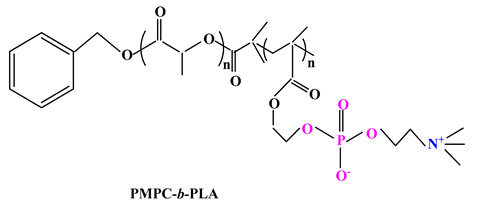	[92]
2	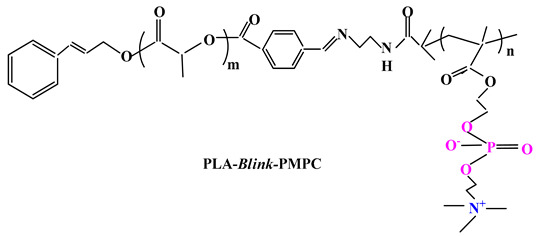	[94]
3	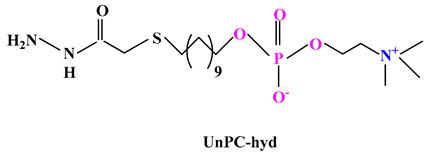	[96]
4	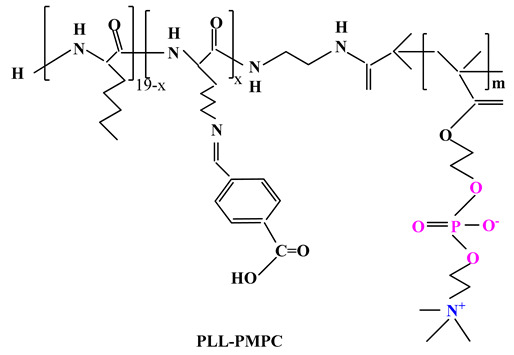	[97]
5	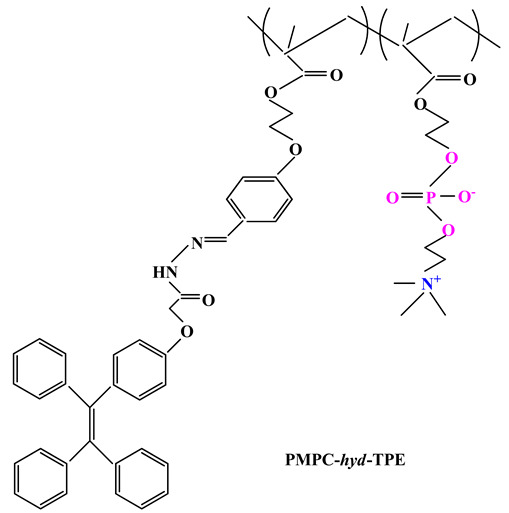	[98]
6	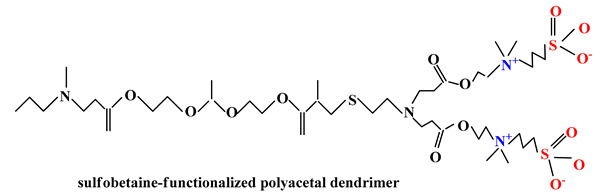	[99]
7	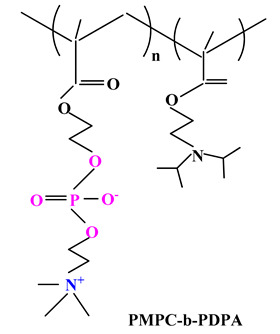	[109]

Folic acid (FA) is known to have a high affinity for the folate receptor (FR) found in the membrane of malignant cells such as ovarian, cervical and nasopharyngeal carcinomas [111]. Therefore, the use of FA transporters could induce cellular uptake of FR-positive cancer cells. For this reason, Armes and colab. [112] continued their studies by directing them towards FA conjugation at the primary amine end of PMPC-PDPA. They initially prepared PPMPC-PDPA by ATRP using 9-fluor-enylmethyl chloroformate (Fmoc) as initiator and then treated purified Fmoc-MPC-DPA with 5-amino-1-pentanol,2-bromoisobutyryl bromide, 1,8-diazabicyclo (5.4.0) undecen-7-ene (DBU) 5% in methanol to obtain H_2_N-MPC-DPA functionalized with the primary amine [113]. FA-MPC-DPC block copolymers with different DPA block lengths were used to prepare pH-sensitive copolymer micelles for the delivery of two antitumor drugs, tamoxifen and PTX.

Tamoxifen was loaded into FA-MPC30-DPA50 micelles while PTX was encapsulated in FA-MPC30-DPA80 micelles. Cell viability studies demonstrated that both drug-loaded micelles were more toxic to tumor cells in an acidic environment. Due to the protonation of the DPA block, the micelles dissociate at pH 5.5, therefore triggering rapid release of the loaded drug. In contrast, minimal cytotoxicity was observed at pH 7.4 because drug-loaded micelles remain intact at this pH of the solution.

Arginyl-glycyl-aspartic acid (RGD) is an oligopeptide responsible for the adhesion of cells to the extracellular matrix (ECM) [114]. RGD can recognize and bind to the α class of integrins, which are overexpressed during tumor growth and therefore are considered as tumor markers [115]. For this reason, RGD is used as a specific target ligand being introduced into various tumor-targeted drug delivery systems.

Dong and colab. [116] have synthesized nanoparticles for antitumor drug delivery. The functional combination of RGD (for active tumor targeting), PCB (for prolonged systemic circulation) and PDPA (for intracellular acid-triggered release) was achieved in order to obtain the RGD-PCB-b-PDPA copolymer via reversible addition−fragmentation chain transfer (RAFT) polymerization followed by functionalization with RGD. DOX was encapsulated in RGD-PCD nanoparticles as a model drug (RGD-PCD/DOX NPs). In vivo and in vitro results demonstrated that RGD-PCD NPs represent a flexible approach to design an antitumor drug delivery system.

Another effective antitumor drug delivery system for improving clinical chemotherapy has been reported by Chen and colab. [117]. They obtained cross-linked biodegradable betaine micelles c(RGDyK) that were prepared from a copolymer of PCBMA, poly(ε-caprolactone) (PCL) and poly(S-2-hydroxyethyl-O-ethyl dithio-carbonate methacrylate) (PSOMA) synthesized by two-step ATRP polymerization. Stability in different media, drug encapsulation efficiency, in vitro release profiles and anticancer efficacy, as well as the ability of c(RGDyK) micelles to target Bcap cells in vitro were studied. The blood circulation and antitumor effect of DOX-loaded c(RGDyK) micelles was also investigated in mice.

These drug delivery systems used in cancer therapy can be administered by the parenteral route.

#### 2.3.2. Other Drug Delivery Systems

Novel pH-responsive biodegradable biomimetic nanocarriers were prepared by Wu et colab. [118] by the self-assembly of N-acetyl-L-histidine-phosphorylcholine-chitosan conjugate (NAcHis-PCCs), which was synthesized by the Atherton-Todd reaction. The PC coating provided antifouling property and excellent biocompatibility to avoid adverse biological responses such as cytotoxicity, haemolysis and complement activation. NAcHis-PCC nanoparticles were loaded with Quercetin as a model drug. Quercetin is a natural flavonoid found abundantly in vegetables and fruits that shows beneficial health effects, being recommended for maintaining immune, respiratory, cardiovascular system health, reducing oxidative stress, inflammation and allergic manifestations. Release studies have shown that the release efficiency of Quercetin was improved in acidic conditions (pH 5.5) as compared to physiological conditions (pH 7.4) leading to the conclusion that NAcHis-PCC nanoparticles can be used successfully as drug delivery systems.

Another oral delivery system consisting of PSBMA and pH-responsive PDPA was synthesized by free radical polymerization by Chen and Wang [119]. This copolymer can self-aggregate into nanoparticles by electrostatic attraction between the ammonium groups and sulfate groups belonging to the PSBMA. The obtained P(SBMA-co-DPA) nanoparticles were loaded with curcumin which is used in recent years as a drug due to its excellent properties (antioxidant, anti-inflammatory, antibacterial, anticancer). The results demonstrated that P(SBMA-co-DPA) nanoparticles can be used as an oral drug delivery system due to the effective release of the loaded drug in the intestine preventing drug damage in the stomach.

Jiang and colab. [120] reported that the polycarboxybetaine coating on a protein effectively improves protein stability, expands inadequate pharmacokinetic (PK) while reducing the immune response. PCB-encapsulated urease exhibited exceptional stability and longer shelf life. In addition, PK behaviour was unchanged and neither anti-uricase nor anti-PCB antibodies were detected after three weekly injections in a rat model. This technology may be applicable to a variety of proteins and lead to the possibility of adopting highly immunogenic proteins for therapeutic or protective applications.

**Figure 5:**
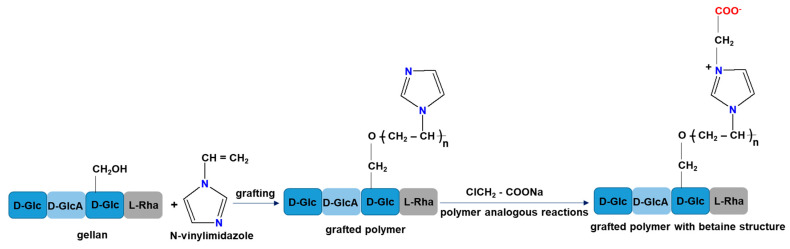
Synthesis routes of grafted polymer with betaine structure.

A new sustained drug release system based on graft copolymers with a betaine structure has been synthesized from gellan and N-vinylimidazole [121]. Graft copolymers with betaine structure were obtained by a two-step reaction (Figure 5). In the first step N-vinylimidazole is grafted onto gellan and the second step is represented by the betainization reaction of the grafted copolymer (PG) with the highest grafting yield in the presence of sodium monochloroacetate (PGB1).

In vitro cefotaxime sodium salts (CF) release studies demonstrated the ability of the new graft copolymers to immobilize amphoteric drugs. It was also revealed that the mechanism of CF release from PG and PGB1 samples was controlled by the diffusion process or a combination of diffusion and swelling processes [121].

The same group of researchers also performed immobilization studies of a 3-(benzoxazole-2′–yl-mercapto-methyl)-4-(p-methoxyphenyl)-5-mercapto-1,2,4-triazole on the same grafted copolymers [122]. The immobilization studies showed that triazole sorption was spontaneous and favored by the increase in temperature. The betaine copolymer incorporated a higher amount of biologically active principle compared to the grafted copolymer without betaine structure. In addition, the mechanism of triazole release from PG and PGB1 copolymers is more complex, being controlled by both swelling and diffusion processes. These results demonstrated that the graft copolymer with a betaine structure may be a potential candidate for the development of oral drug delivery systems as tablets or capsules [122].

Another study was directed towards microparticles based on complexes between chitosan and two poly(N-vinylimidazole)-based polycarboxybetaines that were synthesized by the complex coacervation method. Chloramphenicol succinate sodium salt (CPh) drug retention capacities were higher in the case of CH-PCB complex microparticles than in the case of chitosan microparticles. Based on the release curves it was concluded that CH-PCB complex microparticles can be used as oral suitable drug delivery systems [123].

### 2.4. Wound Healing

A wound is an injury to body tissue that can be caused accidentally (blow, cut, burn) or surgically and can occur with or without tissue loss. Usually, regardless of the type of wound or the amount of tissue lost, wound healing is a complex process that goes through three phases: the cleansing phase, the granulation phase (tissue formation) and the epithelialization phase. Various cells (platelets, lymphocytes, neutrophils, macrophages and fibroblasts) together with growth factors (platelet derived growth factor (PDGF), transforming growth factor-b (TGF-b), basic fibroblast growth factor (bFGF) and epidermal growth factor (EGF) and extracellular matrix proteins play an important role in wound healing [124].

Polybetaines are used to coat other materials where the base material dictates the overall mechanical properties of the device and the polybetaine coating determines the surface properties. Polybetaine surfaces have been shown to be protein-repellent, cell-compatible, non-homolytic and non-thrombogenic. A promising strategy for treating skin wounds is the development of biocompatible polymers in combination with specific drugs or growth factors.

Research has shown that in a moist environment cells and growth factors play an effective role in wound healing because these cells can easily migrate to the wound surface. Moist dressings have the advantage that they can mimic the in vivo environment by facilitating denitrification of necrotic tissue, improving tissue regeneration and avoiding scabbing. It is therefore important that wound dressings are high performance products that can improve the regeneration of damaged skin. Other important factors such as immunogenicity, stability under storage and use conditions and sterilizability must also be considered. It is known that, in general, medical products must have a shelf life of maximum of 5 years, be hydrolytically and thermally stable under conditions of use and above all must be serializable.

Research onto materials with betaine structures has shown that they exhibit in vitro antifouling properties and in vivo anti-inflammatory properties.

**Figure 6:**
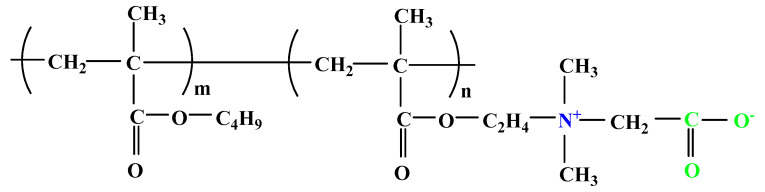
Chemical structure of poly(CMB-r-BMA).

Kitano and colab. [125] reported that a copolymer (poly(CMB-r-BMA)) composed of a carboxybetaine monomer [1-carboxy-N,N-dimethyl-N-(2′-methacryloyloxyethyl) methanaminium inner salt] (CMB) and n-butyl methacrylate (BMA) shows good blood compatibility. Based on this finding, this group of researchers continued their studies following the effect of a thin film of poly(CMB-r-BMA) betaine copolymer (Figure 6) on the healing of excisional and incisional wounds of hairless rats [126].

The results were compared with those obtained using poly(n-butyl methacrylate) (PBMA) and poly(ethylene terephthalate) (PET) films. Thus, it was found that by using the polybetaine films the amount of protein and neutrophils that adhere to this film was low and the excisional wounds healed a few days faster. It was also found that the poly(CMB-r-BMA) film did not improve the healing of the incisional wound. In conclusion, poly(CMB-r-BMA) film shows the potential to be used as a wound dressing.

A number of studies have been directed towards the use of betaine hydrogels as potential wound dressings for the treatment of cutaneous wounds. Betaine hydrogels exhibit strong hydration, excellent antifouling properties, have a biomimetic nature, good biocompatibility and show negligible interactions with proteins and cells making them ideal for the design of wet wound dressings [127,128,129].

Zhang and colab. [127] conducted a comparative study between a hydrogel based on poly (2-hydroxyethyl methacrylate (PHEMA), a commercial wound dressing (DuoDerma) and two betaine hydrogels based on N-(3-sulfopropyl)-N-(methacryloxyethyl)-N,N-dimethylammonium betaine and carboxybetaine acrylamide (CBAA) in order to select the ideal candidate for the treatment of a third-degree burn. It was found that the betaine hydrogels, in particular the polycarboxybetaine-based hydrogels healed the skin wounds better than those treated with the PHEMA hydrogel or the commercial product. It was also observed that betaine hydrogels can be covered with cotton gauze or bandage pads allowing for easier handling. Thus, these studies have demonstrated that PCB and SBMA hydrogels are effective in healing skin wounds and can be used for the development of next generation dressings.

### 2.5. Implant Coatings

An implant is a medical device manufactured to replace a missing biological structure, to help a damaged biological structure or to reinforce an existing biological structure. Cardiac pacemakers, vascular grafts, dental implants, intraocular lenses, central venous catheters, etc. are implants that are placed in specific locations and have a different duration of use.

In general, the immune system of the body recognizes implants as foreign bodies and encapsulates them with a dense collagen capsule [130]. These collagen capsules that form on all implants are impermeable to most molecules in the surrounding microenvironment and inhibit the function of the implant and can cause tissue distortion and pain [131]. To ensure the success of implantable devices, the resistance to protein attachment also prevents triggering the immune response, thus endowing a longer life span for the device.

Taking these considerations into account, Jiang and colab. [132] synthesized two hydrogels, namely PCBMA based on carboxybetaine methacrylate (CBMA) and PHEMA hydrogel using HEMA and poly(ethylene glycol). PCBMA and PHEMA hydrogels were implanted subcutaneously in C57BL/6 mice and the inflammatory response to the implants was evaluated. It was observed that the collagen capsule was formed at 3 months after hydrogel was implanted in mice, PCBMA facilitating the microvessel formation in the surrounding tissue. These essential merits for in vivo applications offer the possibility to improve current medical devices such as glucose sensors and drug delivery devices, tissue scaffolds and artificial organs where fibrotic reactions are undesirable [132].

Polybetaine materials have also found applications as coating surface of a cardiovascular device. Ji and colab. [133] prepared a copolymer based on 2-methacryloyloxyethylphosphorylcholine, stearyl methacrylate, hydroxypropyl methacrylate and trimethoxysiloylpropyl methacrylate via free radical polymerization. The copolymer can be used both as coating of cardiovascular device providing good biocompatibility with blood or as a controlled drug delivery system [133].

Since gold is a biocompatible and corrosion-resistant metal it is very popular in the biomedical field. Coating gold surfaces for medical diagnosis, tissue engineering and drug delivery has been successfully obtained by surface-initiated atom transfer radical polymerization. Poly(carboxybetaine methacrylate) brushes were grafted on a gold surface, and the zwitterionic layer measured 10–15 nm in thickness. By Surface Plasmon Resonance (SPR) spectroscopy, the coated gold surface proved a high resistance to all three proteins tested, which were lysozyme, fibrinogen and human chorionic gonadotropin (hCG). Furthermore, by immobilization of hCG’s monoclonal mouse antibody, the surface became able to selectively bind the target protein while maintaining high resistance to nonspecific protein adsorption (less than 0.3 ng/cm^2^) [102].

Another example of phosphobetaine used as antifouling coating is 2-methacryloyloxyethyl phosphorylcholine telomer deposited as a self-assembled monolayer on a gold electrode, granting high resistance against protein adsorption [134]. In addition, films of poly((3-(methacryloylamino)propyl)-dimethyl(3-sulfopropyl)ammonium hydroxide) (PMPDSAH) were synthesized by surface-initiated ATRP on a gold surface. The amount of nonspecifically adsorbed proteins, determined by SPR was less than 0.6 ng/cm^2^ [135].

Both PMPDSAH and PCBMA layers are comparable with PEG-like film in terms of reducing proteins adsorption. PCBMA layers 10–15 nm thick displayed the best nonspecific resistance, close to the detection limits of some devices of approximatively 3 pg/mm^2^ [30]. These coatings offer an effective method to turn a wide variety of surfaces into nonfouling, biocompatible ones, with high interest in the biomedical field.

Some other examples of polybetaines suitable for biomedical use is the copolymer poly (carboxybetaine acrylate–co–dopamine methacrylate) (PCBDA) which was successfully applied to the surface of a poly(L-lactide) stent. PCBDA was cross-linked with polydopamine (PDA) and polyethyleneimine (PEI), which were co-deposited on the stent surface before adding the polyzwitterion. In vivo tests showed significant anticoagulating, anti-inflammatory and anti-proliferation capabilities [136]. In addition, poly[2-(methacryloyloxyethyl) phosphoryl choline] was used to treat Titanium (Ti) surfaces against protein adsorption, which can trigger various coagulation pathways. The Ti-PMPC surface facilitated the proliferation of MC3T3-e1 cells as long as bone marrow mesenchymal cells (BMSCs) and has great potential in bone tissue engineering [137].

Similarly to the osteoarticular implantable devices, blood-contacting devices such as medical catheters are susceptible to unspecific protein adhesion which leads to premature failure. For example, the polyurethane catheter was coated with a PSBMA based polymer, which provided good surface wettability and resistance to nonspecific protein adsorption [138]. As a result of its capabilities, PCBMA was used as a coating for implantable electrochemical glucose biosensors, to endow biocompatibility and protein adsorption resistance while preserving the sensitivity and linearity of the sensor [139].

## 3. Outlook

The field of polybetaines focused initially on the synthesis, properties and applications in industrial field, such as, sorbents, oil recovery agents, fungicides, flame retardant polymers, emulsifying agents, wetting agents in chemical cleaners, drying agents in the textile industry and cryoprotectants [16,140,141,142,143]. However, in the last 20 years the applications have moved towards biomedical applications, especially in certain areas mentioned in the previous section of this paper. The use of polybetaines against various pathogens as well as against fouling was inspired from the nature. It is well known that the external surface of mammalian cell membrane, in particular the extracellular side of the lipid double layer is rich in phospholipids bearing phosphatidylcholine head groups [144]. The new trends in antimicrobial polybetaines are the synthesis of polymeric materials possessing irreversible and reversible switches, offering the possibility to obtain the smart vehicles for gene and antimicrobial drugs as well as to study the interaction with biological species as protein, DNA, bacteria or human plasma. The functional switches are possible due to the unique properties of polybetaines such as, antipolyelectrolyte effect and upper critical solution temperature (UCST) [17]. Starting from phosphorylcholine, drug delivery systems with complex structures and increased efficiency (vesicles, mice, brushes) have been developed for use mainly in the treatment of cancer. As far as drug delivery systems, studies are in their early stages, because an important part it is still under research such as the simplifying of processes for the synthesis and thus reducing the costs of their mass production. A drawback of the entire process is the fact that the clinical trials take a long time before the product reach the commercial usage. The introduction of charge bioactive molecules in the polymeric chain is a method less exploited at this moment but may become an interesting approach for the future to develop new polymeric materials with betaine moieties and special properties.

## 4. Conclusions

Polybetaines represent a fascinating class of zwitterionic materials that comprise a wide range of structures and properties. Progress in the design and development of materials with betaine structure has a significant impact on the elucidation of their properties as well as on the potential of their practical applications, especially in medical and pharmaceutical fields. In medical applications the development of high performance zwitterionic materials that combine antifouling and antimicrobial properties has become a priority due to the increase in the prevalence of nosocomial infections as well as due to the appearance of new pathogens sometimes quite aggressive.

In this context, designing and optimizing the structure of polybetaines (brushes, nanofibers, nanoparticles, vesicles and micelles) can lead to the obtaining of polymeric materials that act as biocidal polymers or as antimicrobial coatings, capable to prevent bacterial adhesion and biofilm formation, two properties very important in wound healing and implant applications. The studies presented in this review have shown that polybetaines due to their properties such as, antifouling and antimicrobial properties, low cytotoxicity, high biocompatibility, negligible immunogenicity, in vivo stability and long circulation time, represent important candidates in the formulation of various controlled drug delivery systems.

Unfortunately, there are just a few polybetaine materials that are in commercial use, such as Omafilcon A contact lenses for patients with dry eye syndrome. Currently, the percentage of articles regarding polybetaines with biomedical applications is small however it is increasing every year, which shows that this is a field that is in its infancy and can be developed in the future due to the special properties of polybetaines.

## Figures and Tables

**Table 1:** Structures of betainic monomers and polybetaines with antifouling properties.

	Name and Chemical Structures			Ref.
	Sulfobetaines Monomers			
1	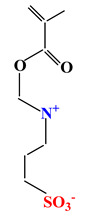	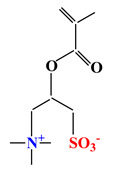	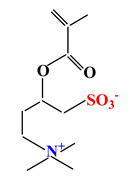	[33]
	**P4VPPC-co-PDMAPC copolymer**			
2	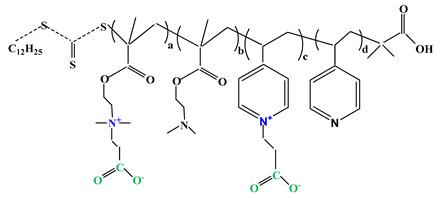			[34]
	**Polybetaines based on poly(ethylene glycol)**			
3	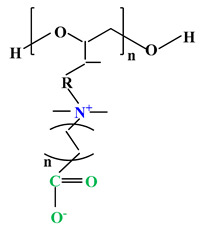	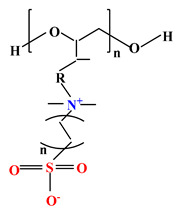	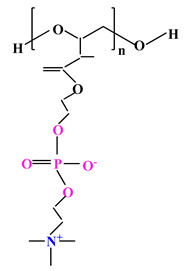	[35]
	**Polybetaines based on poly(acrylates) (PA)**			
4	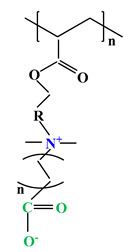	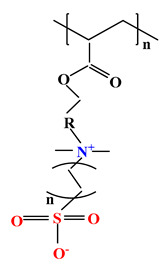	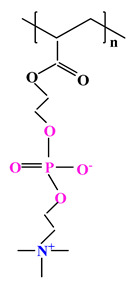	[35]
	**Polybetaines based on poly(acrylamides) (PAA)**			
5	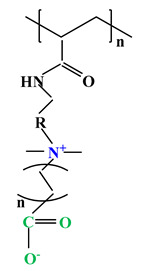	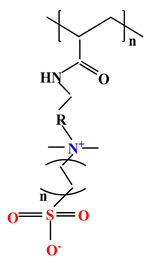	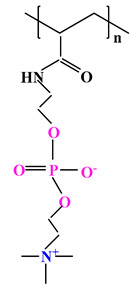	[35]
	**Sulfobetaine-containing terpolymer**			
6	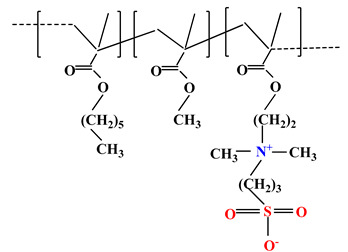			[36]

**Table 2:** The structure of most representative polybetaines used in drug delivery systems.

	Chemical Structure	Name
1	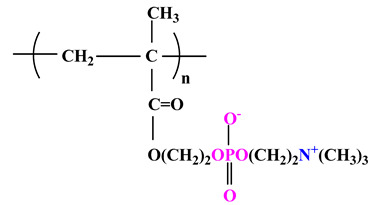	Poly(2-methacryloyloxyethyl phosphorylcholine) (PMPC)
2	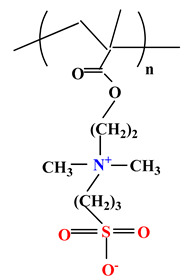	Poly(sulfobetaine methacrylate) (PSBMA)
3	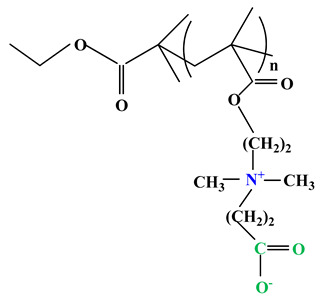	Poly(carboxybetainem methacrylate) (PCBMA)

## Data Availability

Not applicable.

## References

[1] Cao B., Tang Q., Cheng G. (2014). Recent advances of zwitterionic carboxybetaine materials and their derivatives. J. Biomater. Sci. Polym. Ed..

[2] Lowe A.B., McCormick C.L. (2002). Synthesis and solution properties of zwitterionic polymers. Chem. Rev..

[3] Li M., Zhuang B., Yu J. (2020). Functional zwitterionic polymers on surface: Structure and applications. Chem. Asian J..

[4] Blackman L.D., Gunatillake P.A., Cass P., Locok K.E.S. (2019). An introduction to zwitterionic polymer behavior and applications in solution and at surfaces. Chem. Soc. Rev..

[5] Stubbs C., Bailey T.L., Murray K., Gibson M.I. (2020). Polyampholytes as emerging macromolecular cryoprotectants. Biomacromolecules.

[6] Luca C., Neagu V., Vasiliu S., Barboiu V., Dragan S. (2005). Synthetic polybetaines: Synthesis and properties. Focus in Ionic Polymers.

[7] Kudaibergenov S., Jaeger W., Laschewsky A. (2006). Polymeric betains: Synthesis, characterization and application. Adv. Polym. Sci..

[8] Laschewsky A. (2014). Structures and synthesis of zwitterionic polymers. Polymers.

[9] Xue W., Huglin M.B., Liao B. (2006). Observations on the swelling charcateristics of the zwitterionic hydrogel of poly(1-3-sulfopropyl)-2-vinyl-pyridinium-betaine hydrogel. Eur. Polym. J..

[10] Mertoglu M., Laschewsky A., Skrabania K., Wieland C. (2005). New water soluble agents for reversible addition-fragmentation chain transfer polymerization and their application in aqueous solutions. Macromolecules.

[11] Luca C., Mihailescu S., Popa M. (2002). Polymers containing quaternary ammonium groups based on poly(N-vinylimidazole). Eur. Polym. J..

[12] Guo Y.S., Mi Y.F., Ji Y.L., An Q.F., Gao C.J. (2019). One-step surface grafting method for preparing zwitterionic nanofiltation membrane via in situ introduction of initiator in interfacial polymerization. ACS Appl. Polym. Mater..

[13] Yu H.Y., Kang Y., Liu Y., Mi B. (2014). Grafting polyzwitterions onto polyamide by click chemistry and nucleophilic substitution on nitrogen: A novel approach to enhance membrane fouling resistance. J. Membr. Sci..

[14] Cooper B.M., Chan-Seng D., Samanta D., Zhang X., Parelkar S., Emrick T. (2009). Polyester-*graft*-phosphorylcholine prepared by ring- opening polymerization and clik chemistry. Chem. Commun..

[15] Higaki Y., Inutsuka Y., Sakamaki T., Terayama Y., Takenaka A., Higaki K., Yamada N.L., Moriwaki T., Ikemoto Y., Takara A. (2017). Effect of charged group spacer length on hydration state in zwitterionic poly(sulfobetaine) brushes. Langmuir.

[16] Tarannum N., Singh M. (2013). Advances in synthesis and applications of sulfo and carbo analogues of polybetaines. A review. Rev. Adv. Sci. Eng..

[17] Ilcikova M., Tkac J., Kasak P. (2015). Switchable materials containing polyzwitterion moieties. Polymers.

[18] Xiao S., Ren B., Huang L., Shen M., Zhang Y., Zhong M., Yang J., Zheng J. (2018). Salt-responsive zwitterionic polymer brushes with anti-polyelectrolyte property. Curr. Opin. Chem. Eng..

[19] Ladenheim H., Morawetz H. (1957). A new type of polyampholyte: Poly(4-vinyl pyridine betaine). J. Polym. Sci..

[20] Hart R., Timmerman D. (1958). New polyampholytes: The polysulfobetaines. J. Polym. Sci..

[21] Banerjee I., Pangule R.C., Kane R.S. (2011). Antifouling coatings: Recent developments in the design of surfaces that prevent fouling by proteins, bacteria, and marine organisms. Adv. Mater..

[22] Herrwerth S., Rosendahl T., Feng C., Fick J., Eck W., Himmelhaus M., Dahint R., Grunze M. (2003). Covalent coupling of antibodies to self-assembled monolayers of carboxy-functionalized poly(ethylene glycol):  protein resistance and specific binding of biomolecules. Langmuir.

[23] Jiang S., Cao Z. (2010). Ultralow-fouling, functionalizable, and hydrolyzable zwitterionic materials and their derivatives for biological applications. Adv. Mater..

[24] Ruegsegger M.A., Marchant R.E. (2001). Reduced protein adsorption and platelet adhesion by controlled variation of oligomaltose surfactant polymer coatings. J. Biomed. Mater. Res..

[25] Mi L., Jiang S. (2014). Integrated antimicrobial and nonfouling zwitterionic polymers. Angew. Chem. Int. Ed..

[26] Wu J., Zhao C., Hu R., Lin W., Wang Q., Zhao J., Bilinovich S.M., Leeper T.C., Li L., Cheung H.M. (2014). Probing the weak interaction of proteins with neutral and zwitterionic antifouling polymers. Acta Biomater..

[27] Park J., Nam J., Won N., Jin H., Jung S., Jung S., Cho S.H., Kim S. (2011). Compact and stable quantum dots with positive, negative and zwitterionic surface: Specific cell interactions and non-specific adsorptions by the surface charges. Adv. Funct. Mat..

[28] Leng C., Huang H., Zhang K., Hung H.C., Xu Y., Li Y., Jiang S., Chen Z. (2018). Effect of surface hydratation on antifouling properties of mixed charged polymers. Langmuir.

[29] Tanaka M., Morita S., Hayashi T. (2021). Role of interfacial water in determining the interactions of proteins and cells with hydrated materials. Colloid Surf. B Biointerfaces.

[30] Ladd J., Zhang Z., Chen S., Hower J.C., Jiang S. (2008). Zwitterionic polymers exhibiting high resistance to nonspecific protein adsorption from human serum and plasma. Biomacromolecules.

[31] Tanaka M., Sackmann E. (2005). Polymer-supported membranes as models of the cell surface. Nature.

[32] Wang R.Y., Himmelhaus M., Fick J., Herrwerth S., Eck W., Grunze M. (2005). Interaction of self-assembled monolayers of oligo(ethylene glycol)-terminated alkanethiols with water studied by vibrational sum-frequency generation. J. Chem. Phys..

[33] Schonemann E., Koc J., Aldred N., Clare A.S., Laschewsky A., Rosenhahn A., Wischerhoff E. (2019). Synthesis of novel sulfobetaine polymers with differing dipole orientations in their side chains, and their effects on the antifouling properties. Macromol. Rapid Commun..

[34] Wu C., Zhou Y., Wang H., Hu J. (2019). P4VPmodified zwitterionic polymer for the preparation of antifouling functionalized surfaces. Nanomaterials.

[35] Zhang Y., Liu Y., Ren B., Zhang D., Xie S., Chang Y., Yang J., Wu J., Xu L., Zheng J. (2019). Fundamentals and applications of zwitterionic antifouling polymers. J. Phys. D Appl. Phys..

[36] Heath D.E., Cooper S.L. (2012). Design and characterization of sulfobetaine-containing terpolymer biomaterials. Acta Biomater..

[37] Wang Z., van Andel E., Pujari S.P., Feng H., Dijksman J.A., Smulders M.M.J., Zuilhof H. (2017). water-repairable zwitterionic polymer coatings for anti-biofouling surfaces. J. Mat. Chem. B.

[38] Wang T., Wang Y.Q., Su Y.L., Jiang Z.Y. (2006). Antifouling ultrafiltration membrane composed of polyethersulfone and sulfobetaine copolymer. J. Membr. Sci..

[39] Chen S.H., Chang Y., Lee K.R., Wei T.C., Higuchi A., Ho F.M., Tsou C.C., Ho H.T., Lai J.Y. (2012). Hemocompatible control of sulfobetaine-grafted polypropylene fibrous membranes in human whole blood via plasma-induced surface zwitterionization. Langmuir.

[40] Ye S.H., Watanabe J., Iwasaki Y., Ishihara K. (2003). Antifouling blood purification membrane composed of cellulose acetate and phospholipid polymer. Biomaterials.

[41] Paschke S., Lienkamp K. (2020). Polyzwitterions: From surface properties and bioactivity profiles to biomedical applications. ACS Appl. Polym. Mater..

[42] Khan H.A., Baig F.K., Mehboob R. (2017). Nosocomial infections: Epidemiology, prevention, control and surveillance. Asian Pac. J. Trop. Biomed..

[43] Gomez-Vallejo H.J., Uriel-Latorre B., Sande-Meijide M., Villamarin-Bello B., Pavon R., Fdez-Riverela F., Glez-Pena D. (2016). A case-based reasoning system for aiding detection and classification of nosocomial infections. Decis. Supp. Lyst..

[44] Parry B.R., Surovtsev I.V., Cabeen M.T., O´Hern C.S., Dufresne E.R., Jacobs-Wagner C. (2014). The bacterial cytoplasm has glass-like properties and is fluidized by metabolic activity. Cell.

[45] Brock T.D. (1988). The bacterial nucleus: A history. Microbiol. Rev..

[46] Wilson D.N. (2014). Ribosome-targeting antibiotics and mechanisms of bacterial resistance. Nat. Rev. Microbiol..

[47] Kurland C. (1960). Molecular characterization of ribonucleic acid from Escherichia coli ribosomes. J. Mol. Biol..

[48] Meroueh S.O., Bencze K.Z., Hesek D., Lee M., Fisher J.F., Stemmler T.L., Mobashery S. (2006). Three dimensional structure of the bacterial cell wall peptidoglycan. Proc. Natl. Acad. Sci. USA.

[49] Vollmer W., Blanot D., de Pedro M.A. (2008). Peptidoglycan structure and architecture. FEMS Microbiol. Rev..

[50] Huanga K.C., Mukhopadhyayb R., Wena B., Gitaia Z., Wingreena N.S. (2008). Cell shape and cell-wall organization in gram-negative bacteria. Proc. Natl. Acad. Sci. USA.

[51] Song L., Sjollema J., Sharma P.K., Kaper H.J., van der Mei H.C., Busscher H.J. (2014). Nanoscopic vibrations of bacteria with different cell-wall properties adhering to surfaces under flow and static conditions. ACS Nano.

[52] Lemonche L.P., Burns J., Turner R.D., Kumar S., Tank R., Mullin N., Wilson J.S., Chakrabarti B., Bullough P.A., Foster S.J. (2020). The architecture of the Gram-positive bacterial cell wall. Nature.

[53] Beveridge T.J. (1999). Structure of Gram-negative cell walls and their derived membrane vesicles. J. Bacteriol..

[54] Wang L., Hu C., Shao L. (2017). The antimicrobial activity of nanoparticles: Present situation and prospects for the future. Int. J. Nanomed..

[55] Fu M., Liang Y., Lv X., Li C., Yang Y.Y., Yuan P., Ding X. (2021). Recent advances in hydrogel-based anti-infective coatings. J. Mat. Sci. Technol..

[56] Kenawy E.R., Worley S.D. (2007). The chemistry and applications of antimicrobial polymers: State of theart review. Biomacromolecules.

[57] Matsuzaki K. (2009). Control of cell selectivity of antimicrobial peptides. Biochim. Biophys. Acta..

[58] Huang K.S., Yang C.H., Huang S.L., Chen C.Y., Lu Y.Y., Lin Y.S. (2016). Recent advances in antimicrobial polymers: A mini review. Int. J. Mol. Sci..

[59] Francolini I., Donelli G., Crisante F., Taresco V., Piozzi A. (2015). Antimicrobial polymers for anti-biofilm medical devices: State-of-art and perspective. Adv. Exp. Med. Biol..

[60] Ikeda T., Yamaguchi H., Tazuke S. (1984). New polymeric biocides: Synthesis and antibacterial activities of polycations with pendant biguanide groups. Antimicrob. Agents Chemother..

[61] Babutan I., Lucaci A.D., Botiz I. (2021). Antimicrobial polymeric structures assembled on surfaces. Polymers.

[62] Ali A., Jamil M.I., Jiang J., Shoaib M., Amin B.U., Luo S., Zhan X., Chen F., Zhang Q. (2020). An overview of conttrolled-bioacide-release coating based on polymer resin for marine antifouling applications. J. Polym. Res..

[63] Mohanty D., Jena R., Choudhury P.K., Pattnaik R., Mohapatra S., Saini M.R. (2015). Milk derived antimicrobial bioactive peptides: A review. Int. J. Food Prop..

[64] Sanchez A., Vazquez A. (2017). Bioactive peptides: A review. Food Qual. Saf..

[65] Gddoa Al-sahlany S.T., Altemimi A.B., Abd Al Manhel A.J., Niamah A.K., Lakhssasi N., Ibrahim S.A. (2020). Purification et bioactive peptide with antimicrobial properties produced by *Saccharomyces cerevisiae*. Foods.

[66] Hasan J., Crawford R.J., Ivanova E.P. (2013). Antibacterial surfaces: The quest for a new generation of biomaterials. Trends Biotechnol..

[67] Qin Y., Yang H., Xu Z., Li F. (2018). Surface modification of polyacrylonitrile membrane by chemical reactions and physical coating: Comparison between static and pore-flowing procedures. ACS Omega.

[68] Ran B., Jing C., Yang C., Li X., Li Y. (2018). Synthesis of efficient bacterial adhesion-resistant coatings by one-step polydopamine-assisted deposition of branched polyethylenimine-*g*-poly(sulfobetaine methacrylate) copolymers. Appl. Surf. Sci..

[69] Yin X., Liang W., Wang Y., Xiao Y., Zhou Y., Lang M. (2021). Zwitterionic terpolymer based coating potentially for antibacterial and antifouling applications. Mat. Chem. Phys..

[70] Eslamian M., Soltani-Kordshuli F. (2018). Development of multiple-droplet-casting method for the facbrication of coatings and thin solid films. J. Coat. Technol. Res..

[71] Siemann U. (2005). Solvent cast technology-a versatile tool for thin film production. Progr. Colloid Polym. Sci..

[72] Erkoc P., Ulucan-Karnak F. (2021). Nanotechnology-based antimicrobial and antiviral surface coating strategies. Prosthesis.

[73] Park J.H., Choi S., Moon H.C., Seo H., Kim Y.Y., Hong S.P., Lee B.S., Kang E., Lee J., Ryu D.H. (2017). Antimicrobial spray nanocoating of supramolecular Fe(III)-tannic acid metal-organic coordination complex: Applications to shoe insoles and fruits. Sci. Rep..

[74] Kausar A. (2018). Polymer coating technology for high performance applications: Fundamentals and advances. J. Macromol. Sci. Part A.

[75] Hoque J., Akkapeddi P., Yadav V., Manjunath G.B., Uppu D.S., Konai M.M., Yarlagadda V., Sanyal K., Haldar J. (2015). Broad spectrum antibacterial and antifungal polymeric paint materials: Synthesis structure-activity relationship and membrane-active mode of action. ACS Appl. Mater. Interfaces.

[76] Mangal U., Kwon J.S., Choi S.H. (2020). Bio-interactive zwitterionic dental biomaterials for improving biofilm resistance: Characteristics and applications. Int. J. Mol. Sci..

[77] Wei T., Tang Z., Yu Q., Chen H. (2017). Smart antibacterial surfaces with switchable bacteria-killing and bacteria-releasing capabilities. Acs Appl. Mater. Interfaces.

[78] Huang L., Zhang L., Xiao S., Yang Y., Chen F., Fan P., Zhao Z., Zhong M., Yang Y. (2018). Bacteria killing and release of salt-responsive regenerative, double-layered polyzwitterionic brushes. Chem. Eng. J..

[79] Schneider-Chaabane A., Bleicher V., Ran S., Al-Ahmad A., Lienkamp K. (2020). Stimulus-responsive polyxwitterionic surfaces made from itaconic acid: Self-triggered antimicrobial activity, protein repellency and cell compatibility. ACS Appl. Mater. Interfaces.

[80] Zheng L., Sundaram H.S., Wei Z., Li C., Yuan Z. (2017). Applications of zwitterionic polymers. React. Funct. Polym..

[81] Sobolciak P., Spirek M., Katrlik J., Gemeiner P., Lacik I., Kasak P. (2013). Light-switchable polymer from cationic to zwitterionic form: Synthesis, characterization and interactions with DNA and bacterial cells. Macromol. Rapid Coomun..

[82] Cao Q., Mi L., Mendiola J., Ella-Menye J.R., Zhang L., Xue H., Jiang S.Y. (2012). Reversibly switching the function of a surface between attacking and defending against bacteria. Angew. Chem. Int. Ed..

[83] Jia G., Cao Z., Xue H., Xu Y., Jiang S. (2009). Novel zwitterionic-polymer-coated silica nanoparticles. Langmuir.

[84] Yang J., Chen H., Xiao S., Shen M., Chen F., Fan P., Zhong M., Zheng J. (2015). Salt responsive zwitterionic polymer brushes with tunable friction and antifouling properties. Langmuir.

[85] Mi L., Jiang S. (2012). Synchronizing nonfouling and antimicrobial properties in a zwitterionic hydrogel. Biomaterials.

[86] Ward M., Sanchez M., Elasri M.O., Lowe A.B. (2006). Antimicrobial activity of statistical polymethacrylic sulfopropylbetaine against Gram positive and Gram negative bacteria. J. Appl. Polym. Sci..

[87] Horiguchi Y., Goda T., Matsumoto A., Takeuchi H., Yamaoka S., Miyahara Y. (2019). Gold nanoparticles with ligand/zwitterion hybrid layer for individual counting of Influenza A H1N1 subtype using resistive pulse sensing. Langmuir.

[88] Tiwari G., Tiwari R., Srivastava B., Bhati L., Pandey S., Pandey P., Bannerjee S.K. (2012). Drug delivery systems: An update review. Int. J. Pharm. Investig..

[89] Son S., Kim G., Singha K., Park S., Ree M., Kim W.J. (2011). Artificial cell membrane-mimicking nanostructure facilitates efficient gene delivery through fusogenic interaction with the plasma membrane of living cells. Small.

[90] Iwasaki Y., Ishihara K. (2012). Cell membrane-inspired phospholipid polymers for developing medical devices with excellent biointerfaces. Sci. Technol. Adv. Mater..

[91] Konno T., Watanabe J., Ishihara K. (2003). Enhanced solubility of paclitaxel using water-soluble and biocompatible 2-methacryloyloxyethyl phosphorylcholine polymers. J. Biomed. Mater. Res. A.

[92] Hsiue G.H., Lo C.L., Cheng C.H., Lin C.P., Huang C.K., Chen H.H. (2007). Preparation and characterization of poly (2-methacryloyloxyethyl phosphorylcholine)-*block*-poly(D, L-lactide) polymer nanoparticles. J. Polym. Sci. Part A Polym. Chem..

[93] Liu G.Y., Lv L.P., Chen C.J., Liu X.S., Hu X.F., Ji J. (2011). Biocompatible and biodegradable polymersomes for pH-triggered drug release. Soft. Matter..

[94] Ma B., Zhuang W., Liu G., Wang Y. (2018). A biomimetic and pH-sensitive polymeric micelle as carrier for paclitaxel delivery. Regen. Biomater..

[95] Duncan R. (1992). Drug-polymer conjugates: Potential for improved chemotherapy. Anti-Cancer Drugs.

[96] Wang H.B., Liu X.S., Wang Y., Chen Y.J., Jin Q., Ji J. (2015). Doxorubicin conjugated phospholipid prodrugs as smart nanomedicine platforms for cancer therapy. J. Mater. Chem. B.

[97] Ma B., Zhuang W., Wang Y., Luo R., Wang Y. (2018). pH-sensitive doxorubicin-conjugated prodrug micelles with charge-conversion for cancer therapy. Acta Biomater..

[98] Chen Y., Han H., Tong H., Chen T., Wang H., Ji J., Jin Q. (2016). Zwitterionic phosphorylcholine-TPE conjugate for pH-responsive drug delivery and AIE active imaging. ACS Appl. Mater. Interfaces.

[99] Wang Y., Huang D., Wang X., Yang F., Shen H., Wu D. (2019). Fabrication of zwitterionic and pH-responsive polyacetal dendrimers for anticancer drug delivery. Biomater. Sci..

[100] Men Y., Peng S., Yang P., Jiang Q., Zhang Y., Shen B., Dong P., Pang Z., Yang W. (2018). Biodegradable zwitterionic nanogels with long circulation for antitumor drug delivery. ACS Appl. Mater. Inter..

[101] Shao Q., Jiang S. (2015). Molecular understanding and design of zwitterionic materials. Adv. Mater..

[102] Zhang Z., Chen S., Jiang S. (2006). Dual-functional biomimetic materials: Nonfouling poly(carboxybetaine) with active functional groups for protein immobilization. Biomacromolecules.

[103] Ma J., Kang K., Zhang Y., Yi Q., Gu Z. (2018). Detachable polyzwitterion-coated ternary nanoparticles based on peptide dendritic carbon dots for efficient drug delivery in cancer therapy. ACS Appl. Mater. Interfaces.

[104] Ding K., Li R., Ma Y., Li N., Zhang T., Cheng-Mei X., Jiang H.T., Gong Y.K. (2019). Folate ligand orientation optimized during cell membrane mimetic micelle formation for enhanced tumor cell targeting. Langmuir.

[105] Dosio F., Arpicco S., Stella B., Fattal E. (2016). Hyaluronic acid for anticancer drug and nucleic acid delivery. Adv. Drug Deliv. Rev..

[106] Jinrong Peng Q.Y., Xiao Y., Shi K., Liu Q., Hao Y., Yang F., Han R., Qian Z. (2019). Tumor microenvironment responsive drug-dye-peptide nanoassembly for enhanced tumor-targeting, penetration, and photo-chemo-immunotherapy. Adv. Funct. Mater..

[107] Noh T., Kook Y.H., Park C., Youn H., Kim H., Oh E.T., Choi E.K., Park H.J., Kim C. (2008). Block copolymer micelles conjugated with anti-EGFR antibody for targeted delivery of anticancer drug. J. Polym. Sci. Part A Pol. Chem..

[108] Massignani M., LoPresti C., Blanazs A., Madsen J., Armes S.P., Lewis A.L., Battaglia G. (2009). Controlling cellular uptake by surface chemistry, size, and surface topology at the nanoscale. Small.

[109] Giacomelli C., Le Men L., Borsali R., Lai-Kee-Him J., Brisson A., Armes S.P., Lewis A.L. (2006). Phosphorylcholine-based pH-responsive diblock copolymer micelles as drug delivery vehicles: Light scattering, electron microscopy, and fluorescence experiments. Biomacromolecules.

[110] Pegoraro C., Cecchin D., Gracia L.S., Warren N., Madsen J., Armes S.P., Lewis A., MacNeil S., Battaglia G. (2013). Enhanced drug delivery to melanoma cells using PMPC-PDPA polymersomes. Cancer Lett..

[111] Reddy J.A., Low P.S. (1998). Folate-mediated targeting of therapeutic and imaging agents to cancers. Crit. Rev. Ther. Drug Carr. Syst..

[112] Licciardi M., Craparo E.F., Giammona G., Armes S.P., Tang Y., Lewis A.L. (2008). In vitro biological evaluation of folate-functionalized block copolymer micelles for selective anti-cancer drug delivery. Macromol. Biosci..

[113] Licciardi M., Giammona G., Du J.Z., Armes S.P., Tang Y., Lewis A.L. (2006). New folate-functionalized biocompatible block copolymer micelles as potential anti-cancer drug delivery systems. Polymer.

[114] Colombo M., Bianchi A. (2010). Click chemistry for the synthesis of RGD-containing integrin ligands. Molecules.

[115] Sugahara K.N., Teesalu T., Karmali P.P., Kotamraju V.R., Agemy L., Greenwald D.R., Ruoslahti E. (2010). Coadministration of a tumor-penetrating peptide enhances the efficacy of cancer drugs. Science.

[116] Huang P., Song H., Wang W., Sun Y., Zhou J., Wang X., Liu J., Liu J., Kong D., Dong A. (2014). Integrin-targeted zwitterionic polymeric nanoparticles with acid-induced disassembly property for enhanced drug accumulation and release in tumor. Biomacromolecules.

[117] Lin W., Ma G., Kampf N., Yuan Z., Chen S. (2016). Development of long-curculating zwitterionic cross-linked micelles for active-targeted drug delivery. Biomacromolecules.

[118] Wu M., Cao Z., Zhao Y., Zeng R., Tu M., Zhao J. (2016). Novel self-assembled pH-responsive biomimetic nanocarriers for drug delivery. Mater. Sci. Eng. C.

[119] Segregur D., Flanagan T., Mann J., Moir A., Karlsson E.M., Hoch M., Carlile D., Sayah-Jeanne S., Dressman J. (2019). Impact of acid-reducing agents on gastrointestinal physiology and design of biorelevant dissolution tests to reflect these changes. J. Pharm. Sci..

[120] Zhanga P., Suna F., Tsaoa C., Liub S., Jaina P., Sinclaira A., Hunga H.C., Bai T., Wu K., Jiang S. (2015). Zwitterionic gel encapsulation promotes protein stability, enhances pharmacokinetics, and reduces immunogenicity. Proc. Natl. Acad. Sci. USA.

[121] Racovita S., Baranov N., Macsim A.M., Lionte C., Cheptea C., Sunel V., Popa M., Vasiliu S., Desbrieres J. (2020). New grafted copolymers carrying betaine units based on gellan and N-vinylimidazole as precursors for design of drug delivery systems. Molecules.

[122] Baranov N., Racovita S., Vasiliu S., Macsim A.M., Lionte C., Sunel V., Popa M., Desbrieres J., Cheptea C. (2021). Immobilization and release studies of triazole derivatives from grafted copolymer based on gellan-carrying betaine units. Molecules.

[123] Racovita S., Vasiliu S., Vlad C.D. (2010). New drugs delivery systems based on polyelectrolyte complexes. Rev. Roum. Chim..

[124] Tsuboi R., Rifkin D.B. (1990). Recombinant basic fibroblast growth factor stimulates wound healing in healing-impaired db/db mice. J. Exp. Med..

[125] Kitano H., Tada S., Mori T., Takaha K., Gemmei-Ide M., Tanaka M., Fukuda M., Yokoyama Y. (2005). Correlation between the structure of water in the vicinity of carboxybetaine polymers and their blood-compatibility. Langmuir.

[126] Fujishita S., Inaba C., Tada S., Gemmei-Ide M., Kitano H., Saruwatari Y. (2008). Effect of zwitterionic polymers on wound healing. Biol. Pharm. Bull..

[127] Zhu Y., Zhang J., Yang J., Pan C., Xu T., Zhang L. (2016). Zwitterionic hydrogels promote skin wound healing. J. Mater. Chem. B.

[128] He H., Xiao Z., Zhou Y., Chen A., Xuan X., Li Y., Guo X., Zheng J., Xiao J., Wu J. (2019). Zwitterionic poly(sulfobetaine methacrylate) hydrogels with optimal mechanical properties for improving wound healing in vivo. J. Mater. Chem. B.

[129] Asadi N., Pazoki-Toroudi H., Bakhshayesh A.R.D., Akbarzadeh A., Davaran S., Annabi N. (2021). Multifunctional hydrogels for wound healing: Special focus on biomacromolecular-based hydrogels. Int. J. Biol. Macromol..

[130] Langer R. (2009). Perspectives and challenges in tissue engineering and regenerative medicine. Adv. Mater..

[131] Williams D.F. (2008). On the mechanism of biocompatibility. Biomaterials.

[132] Zhang L., Cao Z., Bai T., Carr L., Ella-Menye J.R., Irvin C., Ratner B.D., Jiang S. (2013). Zwitterionic hydrogels implanted in mice resist the foreign-body reaction. Nat. Biotechnol..

[133] Xu J.P., Ji J., Chen W.D., Fan D.Z., Sun Y.F., Shen J.C. (2004). Phospholipid based polymer as drug release coating for cardiovascular device. Eur. Polym. J..

[134] Kitano H., Kawasaki A., Kawasaki H., Morokoshi S. (2005). Resistance of zwitterionic telomers accumulated on metal surfaces against nonspecific adsorption of proteins. J. Colloid Interface Sci..

[135] Cho W.K., Kong B., Choi I.S. (2007). Highly efficient non-biofouling coating of zwitterionic polymers: Poly((3-(methacryloylamino)propyl)-dimethyl(3-sulfopropyl)ammonium hydroxide). Langmuir.

[136] Yang L., Wu H., Liu Y., Xia Q., Yang Y., Chen N., Yang M., Luo R., Liu G., Wang Y. (2022). A robust mussel-inspired zwitterionic coating on biodegradable poly(L-lactide) stent with enhanced anticoagulant, anti-inflammatory, and anti-hyperplasia properties. Chem. Eng. J..

[137] Xu R., Cui X., Xin Q., Lu M., Li Z., Li J., Chen X. (2021). Zwitterionic PMCP-functionalized titanium surface resists protein adsorption, promotes cell adhesion, and enhances osteogenic activity. Colloids Surf. B Biointerfaces.

[138] Zhu Z., Gao Q., Long Z., Huo Q., Ge Y., Vianney N., Daliko N.A., Meng Y., Qu J., Chen H. (2021). Polydopamine/poly(sulfobetaine methacrylate) Co-deposition coatings triggered by CuSO_4_/H_2_O_2_ on implants for improved surface hemocompatibility and antibacterial activity. Bioact. Mater..

[139] Yang W., Xue H., Carr L.R., Wang J., Jiang S. (2011). Zwitterionic poly(carboxybetaine) hydrogels for glucose biosensors in complex media. Biosens. Bioelectron..

[140] Neagu V., Vasiliu S., Racovita S. (2010). Adsorption studies of some inorganic and organic salts on new zwitterionic ion exchangers with carboxybetaine moieties. Chem. Eng. J..

[141] Singh P.K., Singh V.K., Singh M. (2007). Zwitterionic polyelectrolytes: A review. e-Polymers.

[142] Garg G., Chauhan G.S., Ahn J.H. (2011). Polysylfobetaines as extractants for Sr(II) ions from aqueous solutions. Polym. Adv. Technol..

[143] Lloyd A.W., Baker J.A., Smith G., Olliff C.J., Rutt K.J. (1992). A comparison of glycine, sarcosine, N,N-dimethylglycine, glycine betaine and N-modified betaines as liposome cryoprotectants. J. Pharm. Pharmacol..

[144] Schlenoff J.B. (2014). Zwitterion: Coating surfaces with zwitterionic functionality to reduce nonspecific adsorption. Langmuir.

